# Design and Preliminary In Vivo Evaluation of J2H-1802, a Hybrid Compound Derived from 5-ASA and MMF, in a DSS-Induced Colitis Mouse Model

**DOI:** 10.3390/ph19060847

**Published:** 2026-05-29

**Authors:** Myong Jin Lee, Sung-Hoon Park, Gabsik Yang, Jason Kim, Ju Young Lee, Kwanghyun Choi, Kiwon Jung, Ji Hwan Lee, Sumi Lee, Woo-Chan Son, Ki Sung Kang

**Affiliations:** 1 College of Korean Medicine, Gachon University, Seongnam 13120, Republic of Korea; myongene@gachon.ac.kr (M.J.L.); yanggs@gachon.ac.kr (G.Y.); kleert26@gmail.com (J.H.L.); 2 Department of Medical Science, Asan Medical Center, College of Medicine, University of Ulsan, Seoul 05505, Republic of Korea; kuroyume27@gmail.com; 3 J2H biotech Inc., Suwon 16648, Republic of Korea; jsbach@j2hbio.com (J.K.); juyoung105@j2hbio.com (J.Y.L.); ckh@j2hbio.com (K.C.); pharmj@j2hbio.com (K.J.); 4 College of Pharmacy, Seoul National University, Seoul 08826, Republic of Korea; 5 School of Pharmacy, Institute of New Drug Development, Jeonbuk National University, Jeonju 54907, Republic of Korea; chsmath27@jbnu.ac.kr; 6 Department of Pathology, Asan Medical Center, College of Medicine, University of Ulsan, Seoul 05505, Republic of Korea

**Keywords:** J2H-1802, ulcerative colitis, dextran sulfate sodium, inflammatory cytokines, NF-κB, COX-2, colon inflammation, therapeutic agent

## Abstract

**Background/Objectives**: Ulcerative colitis is a chronic inflammatory disorder of the colon characterized by epithelial injury and excessive inflammatory responses. J2H-1802 is a newly synthesized hybrid molecule designed to combine the pharmacological properties of mycophenolate mofetil and 5-aminosalicylic acid. This study evaluated the protective and anti-inflammatory effects of J2H-1802 in a dextran sulfate sodium (DSS)-induced colitis mouse model and investigated its underlying mechanisms. **Methods**: Experimental colitis was induced in mice by administration of 2.5% (*w*/*v*) DSS for 7 days, followed by oral treatment with J2H-1802. Body weight, stool consistency, fecal bleeding, and disease activity index were assessed. Colon length and spleen weight were measured to evaluate macroscopic damage. Levels of tumor necrosis factor-α, interleukin-1β, interleukin-6, and myeloperoxidase in colon tissues were quantified, and the expression of phosphorylated nuclear factor-κB and cyclooxygenase-2 was analyzed by Western blotting. **Results**: J2H-1802 alleviated DSS-induced body weight loss, diarrhea, and fecal bleeding, resulting in reduced disease activity index scores. It also prevented colon shortening and attenuated splenomegaly. In addition, J2H-1802 significantly suppressed the elevated levels of tumor necrosis factor-α, interleukin-1β, interleukin-6, and myeloperoxidase in colon tissues. Western blot analysis further showed that J2H-1802 inhibited the DSS-induced upregulation of phosphorylated nuclear factor-κB and cyclooxygenase-2. **Conclusions**: J2H-1802 protected against DSS-induced colitis by reducing inflammatory responses and inhibiting the nuclear factor-κB/cyclooxygenase-2 signaling pathway. These findings suggest that J2H-1802 functions as a hybrid anti-inflammatory scaffold with in vivo pharmacological activity and may warrant further optimization and investigation in IBD models.

## 1. Introduction

Inflammatory bowel disease (IBD), which includes ulcerative colitis (UC) and Crohn’s disease (CD), is a chronic inflammatory condition of the gastrointestinal tract with a rising global incidence [1,2]. Once considered prevalent mainly in Western countries, IBD is now increasing worldwide, including in Asia and other newly industrialized countries, likely due to globalization and improved communication technologies [3,4]. This increasing trend has raised growing concern about IBD as a major global health issue [4,5]. Among chemically induced animal models of colitis, dextran sulfate sodium (DSS)-induced colitis is commonly employed because of its reliability and flexibility. DSS mimics many pathological and clinical features of human UC, including epithelial barrier disruption, mucosal ulceration, and inflammatory cell infiltration [6,7,8]. In mice, DSS administration damages colonic epithelial cells and increases intestinal permeability, leading to inflammation driven by microbial factors and immune responses [9,10]. Owing to its reproducibility and close resemblance to human disease, the DSS model is widely used to study colitis pathogenesis and to evaluate potential therapeutic interventions [9,10]. Recent studies have focused on identifying novel compounds that can ameliorate DSS-induced colitis by modulating inflammatory pathways, restoring epithelial integrity, or altering gut microbiota composition [9,11]. Given the multifactorial nature of colitis, agents that target both inflammation and barrier function hold strong therapeutic potential [11]. This study aims to evaluate the protective effects of J2H-1802 against DSS-induced colitis in mice by assessing clinical symptoms and inflammatory cytokine expression. Understanding the therapeutic potential and mechanisms of J2H-1802 may contribute to the development of new strategies for managing IBD. Five-aminosalicylic acid (5-ASA) has been the cornerstone therapy for mild-to-moderate UC [12]. Its therapeutic effects are primarily attributed to local anti-inflammatory actions on the intestinal mucosa, including inhibition of COX-2-derived prostaglandins, activation of PPAR-γ, and suppression of NF-κB-mediated cytokine production [13]. Numerous randomized trials and meta-analyses have demonstrated that 5-ASA is superior to placebo for both induction and maintenance of remission in UC, and that once-daily formulations are as effective as conventional divided dosing [14]. In contrast, the clinical benefit of 5-ASA in CD is limited, with evidence suggesting only modest efficacy in selected postoperative or mild colonic phenotypes [14]. Mycophenolate mofetil (MMF), an inhibitor of inosine monophosphate dehydrogenase, has been investigated as an alternative immunomodulatory agent for patients with IBD who are intolerant of or refractory to thiopurines [15]. In the absence of large randomized trials, prospective and retrospective studies have reported that approximately 60–65% of patients with UC or CD achieve clinical remission with MMF, and a substantial proportion maintain remission with continued treatment [15]. These findings suggest that MMF may be a useful option in selected cases of refractory IBD, despite its limited role in standard therapeutic algorithms. J2H-1802 is a novel hybrid compound synthesized by combining structural motifs of MMF and 5-ASA. This design aims to integrate the immunosuppressive and anti-inflammatory activities of MMF with the mucosal-protective and antioxidant properties of 5-ASA. J2H-1802 was designed to integrate structural motifs of 5-ASA and MMF and may provide combined anti-inflammatory properties. This hybridization strategy is intended to achieve targeted anti-inflammatory action in gastrointestinal tissues while minimizing systemic immunosuppression [16].

Although current therapies for UC target a broad range of inflammatory pathways, their efficacy is often limited by incomplete mucosal healing, systemic adverse effects, and insufficient suppression of chronic inflammation [17]. Therefore, developing agents that act locally within the intestinal mucosa while exerting multi-targeted anti-inflammatory effects remains an important unmet need [18]. Unlike conventional combination therapies, which involve the co-administration of multiple drugs, hybrid compounds integrate distinct pharmacophores into a single molecular entity. This strategy may provide potential advantages, including coordinated pharmacological activity, improved bioavailability, and enhanced therapeutic efficacy [16]. In this context, J2H-1802 was designed as a novel hybrid compound combining the structural and functional properties of 5-ASA and MMF.

J2H-1802, a rationally designed hybrid compound, represents a novel therapeutic approach in this context [16]. While its structural framework integrates anti-inflammatory motifs, the biological properties of J2H-1802 have not yet been characterized in experimental models of colitis. In this study, we present the first in vivo evidence that J2H-1802 provided robust protection against DSS-induced colitis. Notably, J2H-1802 mitigated clinical symptoms and tissue damage while exerting dual regulatory effects by suppressing pro-inflammatory cytokine production and inhibiting NF-κB/COX-2 signaling in colonic tissues. These findings highlighted J2H-1802 as a promising candidate capable of overcoming limitations of existing treatments through targeted mucosal anti-inflammatory action and multi-pathway modulation.

## 2. Results

### 2.1. J2H-1802 Improves Body Weight Loss and Disease Activity Index in DSS-Induced Colitis Mice

**Figure 1:**
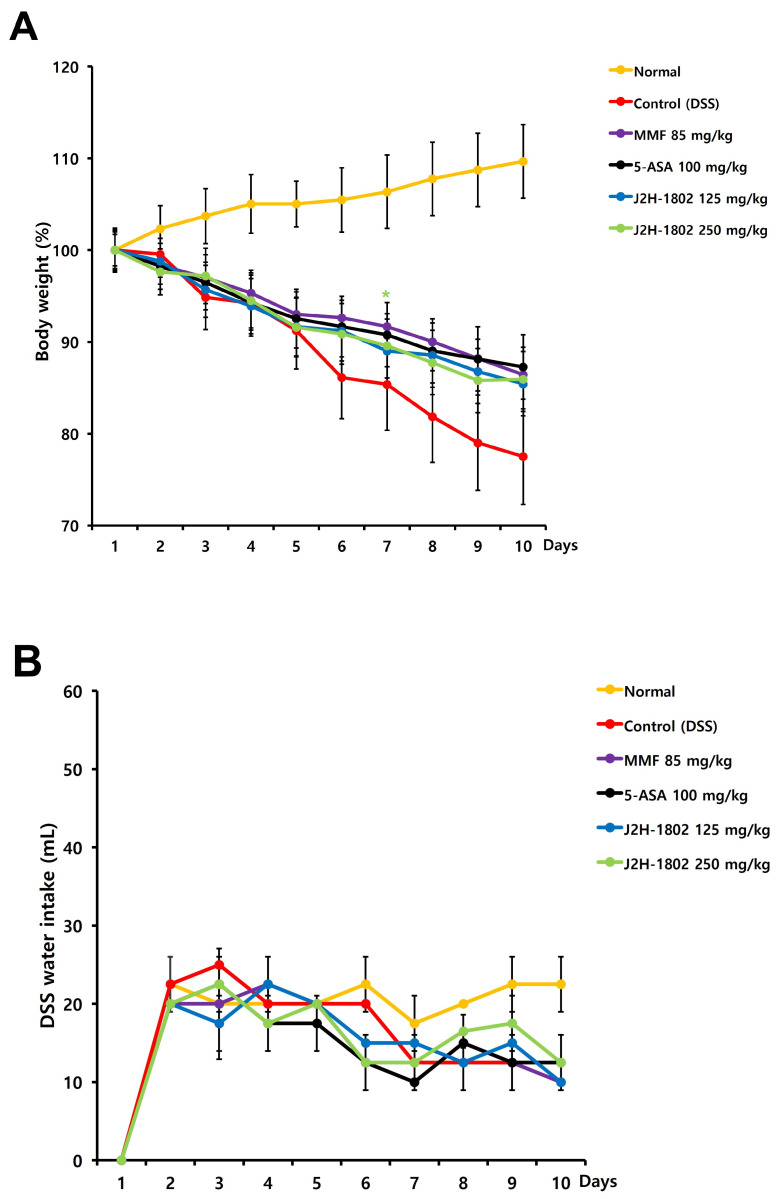

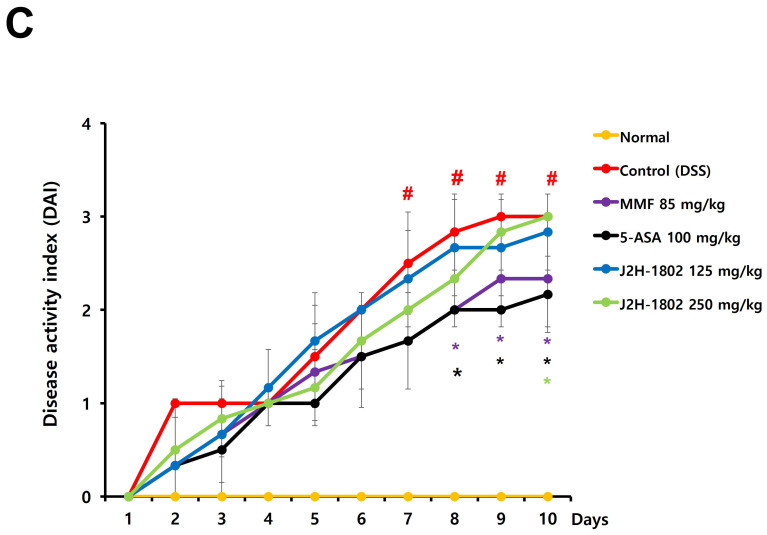
Effects of J2H-1802 on clinical markers in dextran sulfate sodium (DSS)-induced coli tis mice. Acute colitis was induced by administering 2.5% DSS in drinking water for 10 days. J2H-1802 was administered once daily during DSS treatment. (**A**) Body weight, (**B**) DSS water intake, and (**C**) DAI score. Data are presented as mean ± SD (*n* = 6). Statistical significance was evaluated relative to the DSS control group at each time point using two-way repeated measures ANOVA followed by Tukey’s post hoc test. ^#^ *p* < 0.05 versus normal group; * *p* < 0.05 versus DSS control group.

As shown in Figure 1A, body weight decreased in DSS-treated groups during the experimental period, consistent with DSS-induced colitis. Treatment with J2H-1802 (250 mg/kg) partially attenuated DSS-induced body weight loss and showed a significant difference compared with the DSS control group at day 7 (*p* = 0.0462). Figure 1B shows the water intake in each group. DSS-treated groups exhibited a gradual decrease in water consumption compared with the normal group. Figure 1C presents the DAI, calculated by summing the diarrhea and hematochezia scores to assess colitis severity. The DSS group received 2.5% DSS for 10 days to induce acute colitis. Stool consistency began to soften on day 3, with diarrhea appearing from day 6 and persisting until the end of the experiment. Blood was first detected on day 3 after DSS administration, and overt hematochezia appeared on days 4–5. The DSS control group showed significantly elevated DAI scores compared with the normal group during the late phase of colitis progression at multiple time points (*p* < 0.05). Treatment with MMF and 5-ASA significantly attenuated DSS-induced increases in DAI scores compared with the DSS control group (*p* < 0.05). In the J2H-1802-treated groups, stool consistency and hematochezia scores gradually improved during the late stage of DSS administration. In particular, the DAI score from day 7 onward decreased in mice treated with J2H-1802 (250 mg/kg).

### 2.2. J2H-1802 Prevents Colon Shortening and Reduces Spleen Weight in DSS-Induced Colitis Mice

**Figure 2:**
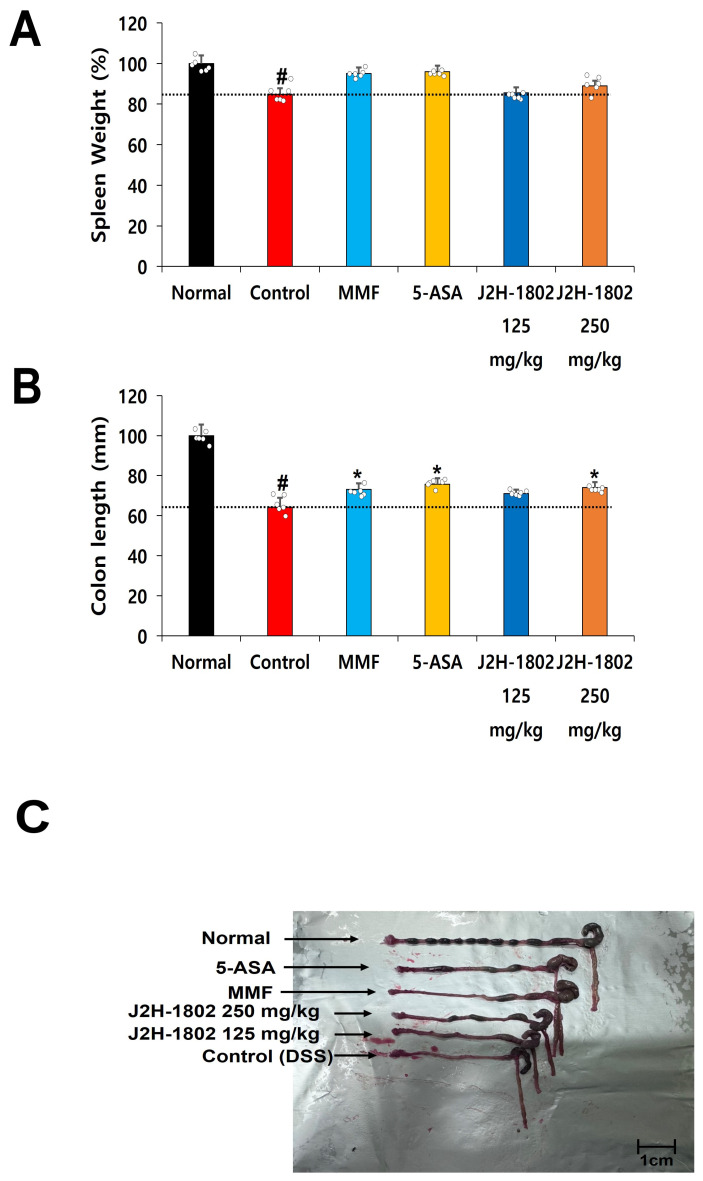
Effects of J2H-1802 on spleen weight and colon length in DSS-induced colitis mice. The disease was induced by administering 2.5% DSS in drinking water for 10 days. J2H-1802 was administered once daily during DSS treatment. (**A**) Spleen weight, (**B**) Quantitative analysis of colon length, and (**C**) Colon length. Data are presented as mean ± SD. Statistical differences were analyzed using one-way ANOVA followed by Tukey’s multiple comparison test. ^#^ *p* < 0.05 versus normal group; * *p* < 0.05 versus DSS control group. The dotted line indicates the reference level of DSS-induced changes.

To assess organ changes associated with DSS-induced inflammation, the spleen and colon were excised after DSS treatment. As shown in Figure 2A, no significant differences in spleen weight were observed between the DSS control group and the J2H-1802-treated groups (125 mg/kg, *p* = 0.0627; 250 mg/kg, *p* = 0.4487). However, MMF treatment significantly restored spleen weight compared with the DSS group (*p* = 0.0028).

Colon shortening—a well-established marker of colitis severity—was evident in the DSS group, with colon length reduced by approximately 36% compared with the normal group. In contrast, J2H-1802 treatment significantly attenuated DSS-induced colon shortening (*p* = 0.0013), whereas the 125 mg/kg group did not show a statistically significant difference compared with the DSS group (*p* = 0.0716). Similar protective effects were observed in the 5-ASA (*p* < 0.0001) and MMF (*p* = 0.0009) groups (Figure 2B,C).

### 2.3. J2H-1802 Suppresses Pro-Inflammatory Cytokine Production in Colon Tissue of DSS-In Duced Colitis Mice

**Figure 3:**
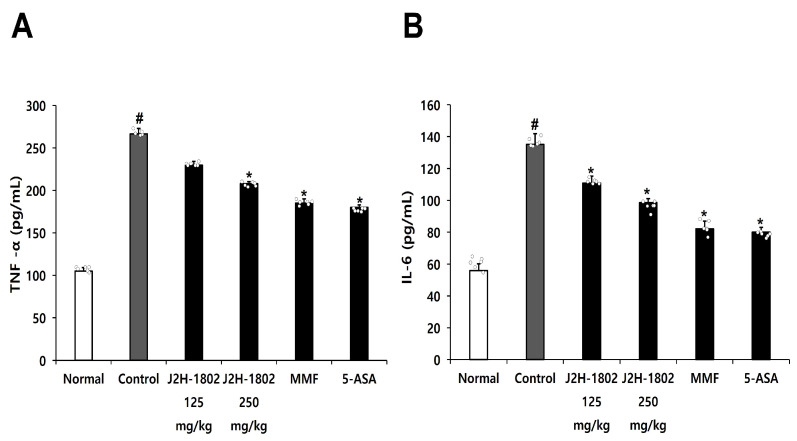

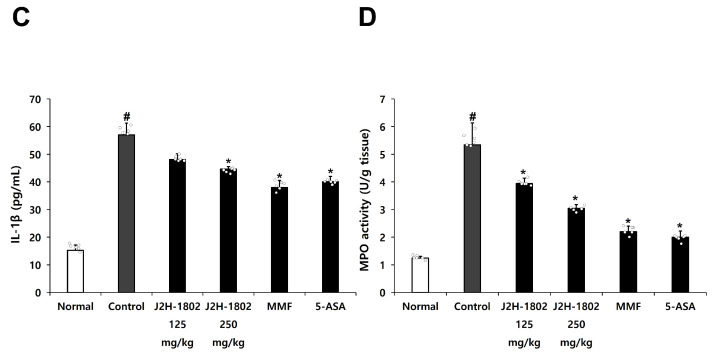
Effects of J2H-1802 on pro-inflammatory cytokines and MPO activity in DSS–induced colitis mice. Acute colitis was induced by administering 2.5% DSS in drinking water for 10 days. J2H-1802 was administered once daily during DSS treatment. (**A**) TNF-α levels in colon tissue. (**B**) IL-6 levels in colon tissue. (**C**) IL-1β levels in colon tissue. (**D**) MPO activity in colon tissue. Cytokine levels are expressed as pg/mL in serum, while MPO activity is expressed as U/mg in colon tissue. Data are presented as mean ± SD. Statistical differences were analyzed using one-way ANOVA followed by Tukey’s multiple comparison test. ^#^ *p* < 0.05 versus normal group; * *p* < 0.05 versus DSS control group.

DSS damages the intestinal epithelial lining, leading to increased production of pro-inflammatory cytokines. To evaluate the anti-inflammatory effects of J2H-1802, the levels of TNF-α, IL-1β, IL-6, and MPO were measured (Figure 3). As shown in Figure 3A, TNF-α levels were markedly reduced by 22% in the J2H-1802 (250 mg/kg) group compared with the DSS group (*p* < 0.0001). In addition, the 250 mg/kg group showed a significantly greater reduction than the 125 mg/kg group (*p* = 0.0033), supporting a dose-dependent effect. DSS-induced elevation of IL-6 was significantly decreased by 28% following treatment with J2H-1802 (250 mg/kg) (*p* < 0.0001) (Figure 3B). However, the difference between the 125 and 250 mg/kg groups was not statistically significant (*p* = 0.1516). IL-1β levels in the DSS group reached 57.0 pg/mL, approximately 3.8-fold higher than those in the normal group (15.2 pg/mL) (Figure 3C). Treatment with J2H-1802 significantly reduced IL-1β levels by 22% compared with the DSS group (*p* = 0.0034). No significant difference was observed between the 125 and 250 mg/kg groups (*p* = 0.7248). DSS administration induced neutrophil infiltration into the colonic mucosa, resulting in increased MPO activity, a widely used marker of neutrophil infiltration and inflammatory activity in colitis models. MPO activity in the DSS group was approximately 4.3-fold higher than that in the normal group, indicating a severe inflammatory response. In contrast, J2H-1802 treatment reduced MPO activity by 43% compared with the DSS group (*p* = 0.0003) (Figure 3D). Although the 250 mg/kg group showed lower MPO activity than the 125 mg/kg group, the difference was not statistically significant (*p* = 0.1805). No statistically significant differences were observed between J2H-1802 (250 mg/kg) and the reference compounds 5-ASA or MMF in the evaluated inflammatory markers under the present experimental conditions.

### 2.4. J2H-1802 Inhibits NF-κB Activation and COX-2 Expression in Colon Tissue of DSS-Induced Colitis Mice

**Figure 4:**
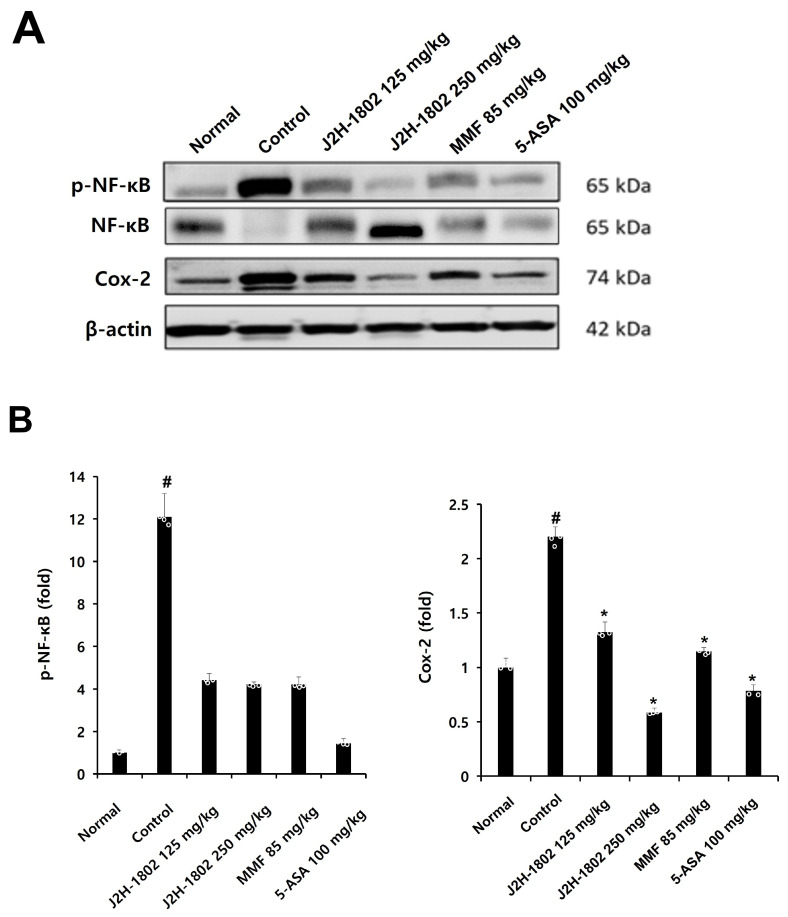
Effects of J2H-1802 on inflammatory signaling pathways in colon tissue from. DSS-induced colitis mice. (**A**) Protein expression of COX-2 and phosphorylation of NF-κB in colon tissue were assessed by Western blotting. (**B**) Relative protein levels were quantified by densitometry. COX-2 density was normalized to β-actin, and phosphorylated NF–κB was normalized to total NF-κB. The normalized values were then expressed relative to the normal group. Data are presented as mean ± SD of three independent pooled samples per group (two mice/sample). Statistical differences were analyzed by one-way analysis of variance (ANOVA) followed by Tukey’s multiple comparison test. ^#^ *p* < 0.05 versus normal group; * *p* < 0.05 compared with the control group.

Protein expression levels of NF-κB, phosphorylated NF-κB (p-NF-κB), and COX-2 were analyzed by Western blotting (Figure 4). DSS administration markedly increased p-NF-κB and COX-2 expression compared with the normal group. In contrast, J2H-1802 treatment significantly reduced DSS-induced upregulation of both COX-2 and p-NF-κB (Figure 4). DSS exposure activated NF-κB phosphorylation in colonic tissues, whereas J2H-1802 attenuated this effect, suggesting inhibition of NF-κB signaling. Notably, J2H-1802 at 250 mg/kg reduced DSS-induced NF-κB phosphorylation, consistent with reduced COX-2 expression.

## 3. Discussion

Current therapies for IBD, including 5-ASA and immunomodulatory agents, remain limited by incomplete efficacy, relapse, and safety concerns [19,20,21,22]. These limitations support the need for novel therapeutic strategies. In this context, the development of a single hybrid molecule integrating the local anti-inflammatory activity of 5-ASA with the immunomodulatory properties of MMF represents a potentially promising approach [22,23,24]. The development of a single hybrid molecule that integrates the local anti-inflammatory activity of 5-ASA with the systemic immunomodulatory effects of MMF represents a potential strategy to address some limitations associated with current therapies [22,23,24]. The hybrid design strategy may offer a pharmacological advantage by integrating two distinct anti-inflammatory pharmacophores into a single molecular scaffold, which could potentially improve therapeutic coordination and reduce variability associated with co-administration of separate agents. The present study demonstrated that J2H-1802—a chemically linked 5-ASA/MMF hybrid—attenuated inflammatory responses in a DSS-induced murine colitis model. Oral administration of J2H-1802 ameliorated DSS-induced weight loss, diarrhea, and rectal bleeding, resulting in a reduction in the DAI. These results suggested that J2H-1802 alleviated inflammatory responses associated with DSS-induced colitis [6,7,10]. The pathogenesis of DSS-induced colitis involves epithelial barrier disruption, immune cell activation, and the release of inflammatory mediators, including TNF-α, IL-1β, and IL-6, with increased MPO activity reflecting neutrophil infiltration. J2H-1802 treatment reduced these cytokine levels and MPO activity, suggesting attenuation of inflammatory responses associated with DSS-induced colitis. These effects are consistent with reports showing that inhibition of these mediators restores epithelial function in colitis models [6,7]. NF-κB is a key regulator of inflammation, driving transcription of COX-2 and pro-inflammatory cytokines. J2H-1802 suppressed DSS-induced NF-κB phosphorylation and COX-2 expression in colon tissue, indicating inhibition of upstream inflammatory signaling events [19,25,26]. Although J2H-1802 attenuated DSS-induced NF-κB phosphorylation and COX-2 expression, its effects were not statistically superior to those observed with the reference compounds under the present experimental conditions. In addition to suppression of NF-κB signaling, J2H-1802 may influence multiple upstream inflammatory pathways involved in DSS-induced colitis. DSS-mediated epithelial injury activates MAPK and JAK/STAT signaling cascades, leading to enhanced transcription of pro-inflammatory mediators and amplification of mucosal immune responses. Given that J2H-1802 reduced the expression of inflammatory cytokines and COX-2, the compound may exert broader regulatory effects on interconnected inflammatory signaling networks. Furthermore, hybrid scaffold design may facilitate simultaneous modulation of multiple molecular targets, potentially contributing to coordinated anti-inflammatory activity. However, these mechanistic possibilities remain speculative and require further experimental validation.

Recent studies on hybrid drug design have demonstrated that integrating multiple pharmacophores into a single molecule can enhance therapeutic efficacy and enable simultaneous modulation of multiple inflammatory pathways. In line with these findings, the present study shows that J2H-1802 effectively attenuates inflammatory responses in a DSS-induced colitis model, supporting the potential of hybridization as a strategy for improving anti-inflammatory therapies. Similar NF-κB-inhibitory effects have been observed for other hybrid compounds, such as diclofenac–eugenol hybrids that mitigate NF-κB-mediated inflammation in vitro and in vivo, and tofacitinib–ADTOH hybrids for UC that exhibit pleiotropic anti-inflammatory activity [27,28]. In the DSS-induced colitis model, J2H-1802 demonstrated measurable anti-inflammatory activity, including attenuation of clinical symptoms and reduction in inflammatory cytokines. The observed anti-inflammatory activity of J2H-1802 in the DSS model suggests that this hybrid scaffold retains pharmacological activity and may warrant further pharmacological investigation. Although J2H-1802 did not demonstrate superior efficacy compared with the reference compounds 5-ASA or MMF under the present experimental conditions, the compound exhibited consistent anti-inflammatory activity in the DSS-induced colitis model and should be considered as a preliminary hybrid scaffold requiring further optimization. In addition, hybrid molecules may offer advantages beyond direct efficacy comparisons, including differences in pharmacokinetics, tissue distribution, metabolic stability, safety profiles, and opportunities for further structural optimization.

The dose range selected for J2H-1802 was based on preliminary tolerability observations and commonly used dosing ranges for small-molecule anti-inflammatory compounds in DSS-induced colitis models, and therefore was not intended to establish direct potency equivalence with the reference compounds. These findings suggest that the hybrid scaffold retains pharmacological activity and may serve as a starting point for further structural optimization and mechanistic investigation. These findings support further investigation of J2H-1802 as a hybrid anti-inflammatory scaffold with potential relevance to inflammatory disorders. However, the precise molecular targets, upstream signaling, intestinal microbiota effects, and pharmacokinetics of J2H-1802 remain to be elucidated [12,13,15,16,17,29]. Taken together, these findings provided preliminary evidence that J2H-1802 exhibits anti-inflammatory activity in a DSS-induced colitis model, potentially associated with reduced cytokine production and modulation of NF-κB signaling. Further studies are needed to clarify its clinical translation potential and applicability in addressing unmet needs in UC. Several limitations of the present study should be acknowledged. First, pharmacokinetic characterization of J2H-1802 was not performed, and therefore its metabolic stability and tissue distribution remain unknown. While this limits a full assessment of its translational potential, the present study demonstrates clear in vivo anti-inflammatory efficacy in a DSS-induced colitis model, providing a strong foundation for further pharmacological evaluation. Second, direct comparative studies with the parent compounds (5-ASA and MMF), including their physical co-administration at equimolar doses, were not included in this study. Therefore, it remains unclear whether the observed effects of J2H-1802 are attributable to chemical hybridization itself or to additive pharmacological interactions between the parent compounds. Future studies incorporating direct combination comparisons will be necessary to clarify the mechanistic advantages of the hybrid design. Third, although the selected doses demonstrated significant efficacy, a broader dose–response analysis would be necessary to further define the optimal therapeutic range of J2H-1802. Fourth, although NF-κB and COX-2 signaling were examined as representative inflammatory pathways, additional mechanistic studies are required to fully elucidate the molecular targets of J2H-1802. Future studies will expand the mechanistic investigation to include additional signaling pathways such as MAPK, JAK/STAT, and oxidative stress-related mechanisms to provide a more comprehensive understanding of the action of J2H-1802. Fifth, histopathological evaluation of colon tissue was not performed, which limits direct assessment of tissue-level protection in the DSS-induced colitis model. While the present study demonstrates consistent improvements in functional and biochemical parameters, inclusion of histological analysis would provide more comprehensive evidence of epithelial protection and inflammatory changes. Further investigation into pharmacokinetics, tissue distribution, and broader signaling pathways will be important to fully elucidate the therapeutic potential of J2H-1802.

## 4. Materials and Methods

### 4.1. Sample Preparation

**Scheme 1:**
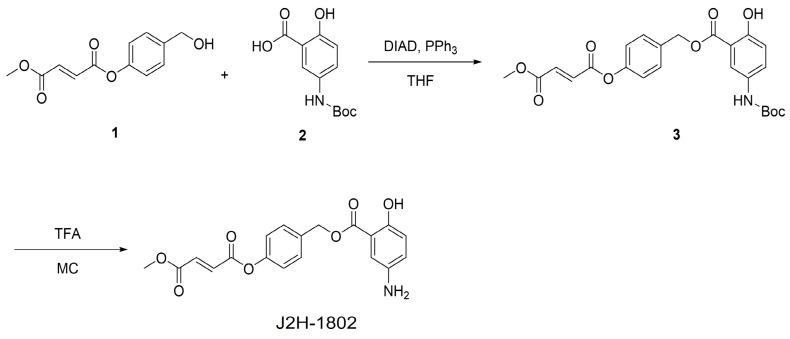
Synthesis of J2H-1802.

**Figure 5:**
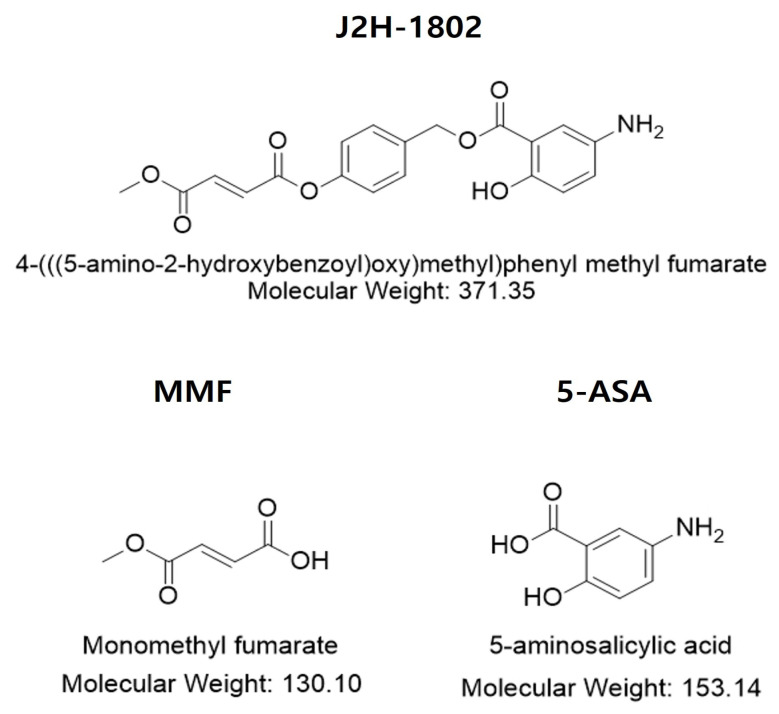
Chemical structure of J2H-1802, a synthetic hybrid compound combining the structural motifs of mycophenolate mofetil (MMF) and Five-aminosalicylic acid (5-ASA).

J2H-1802 was synthesized as described in Scheme 1 and obtained as a white crystalline powder with high purity (>98%), confirmed by high-performance liquid chromatography analysis. J2H-1802 (Figure 5) is a synthetic hybrid compound created by conjugating structural elements of MMF and 5-ASA to combine the immunosuppressive and anti-inflammatory activities of MMF with the mucosal-protective and antioxidant effects of 5-ASA. Its molecular structure was verified by proton nuclear magnetic resonance (^1^H-NMR) and liquid chromatography-mass spectrometry (LC-MS). For experimental use, J2H-1802 was dissolved in dimethyl sulfoxide (DMSO) to prepare a 10 mM stock solution and diluted to the required concentrations in culture medium before use. All other reagents and solvents were of analytical grade. The ^1^H-NMR spectrum and LC-MS data confirming the molecular structure of J2H-1802 are provided in the Supplementary Materials (Figures S1–S3).

### 4.2. Mice

Female BALB/c mice (6–8 weeks old) were housed under controlled temperature (20–23 °C), humidity (~60%), and a 12 h light/dark cycle, with free access to standard chow and water. All procedures were approved by the Institutional Animal Care and Use Committee of Gachon University (GU1-2023-IA0053-00, approved on 27 December 2024) and conducted in accordance with institutional guidelines for the care and use of laboratory animals. Animals were monitored daily for clinical signs including body weight loss, stool consistency, rectal bleeding, posture, and activity. Humane endpoint criteria were defined in accordance with the approved IACUC protocol and included substantial body weight loss (approaching 25%), severe dehydration, or inability to access food or water. Although DSS-treated mice exhibited notable body weight loss, no animals reached the predefined humane endpoint criteria requiring euthanasia, and no unscheduled deaths occurred during the experiment.

### 4.3. DSS-Induced Colitis Mouse Model and Treatment

**Figure 6:**
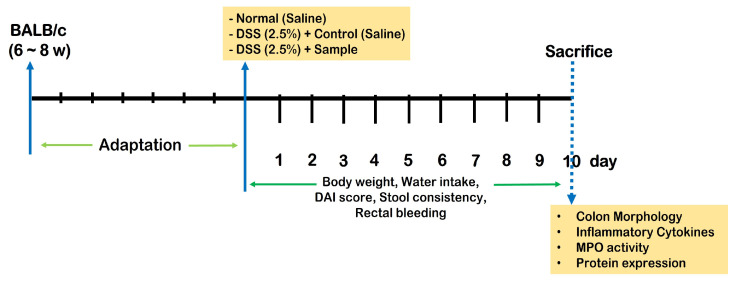
Timeline of the DSS (Dextran Sulfate Sodium)-induced inflammatory bowel disease (IBD) model. Mice were randomly divided into treatment groups including normal, DSS control, 5-ASA, MMF, and J2H-1802 (*n* = 6 per group). J2H-1802 was orally administered in a vehicle of 10% DMSO and 30% HPβCD; control and normal groups received vehicle or saline, respectively.

Chronic colitis was induced by administering 2.5% (*w*/*v*) DSS (MW 36–50 kDa; MP Biomedicals, Santal Ana, CA, USA) in drinking water, alternating daily with regular water to sustain disease progression (Figure 6). After a 7-day acclimation period, mice were randomly assigned to treatment groups including normal, DSS control, 5-ASA, MMF, and J2H-1802 treatment groups (*n* = 6 per group)**.** Importantly, all treatment groups, including DSS control, 5-ASA, MMF, and J2H-1802, were included in the same DSS-induced colitis experiment and evaluated under identical experimental conditions. The vehicle consisted of 10% dimethyl sulfoxide (DMSO) and 30% hydroxypropyl-β-cyclodextrin (HPβCD) diluted in saline. J2H-1802, 5-ASA and MMF were administered orally once daily using this vehicle. Control animals received the vehicle alone, while normal mice received saline. The selected doses of J2H-1802 (125 and 250 mg/kg) were based on preliminary tolerability observations, including the absence of overt toxicity and stable body weight profiles, as well as commonly used dosing ranges for small-molecule anti-inflammatory compounds in DSS-induced colitis models. The doses of comparator compounds 5-ASA (100 mg/kg) and MMF (85 mg/kg) were selected based on previously reported studies using DSS-induced colitis models. The DSS-induced colitis protocol was modified from our previously described method [25].

### 4.4. Assessment of Colitis

During the experiment, body weight (%), water intake, diarrhea, and rectal bleeding were monitored daily. The disease activity index (DAI) was scored daily by two blinded investigators using the criteria in Table 1. No food restrictions were applied to avoid influencing drug response. At the end of the study, mice were euthanized, and the spleen and colon were collected. Spleen weight and colon length were measured, and colon tissues were fixed in 10% formalin or stored at −80 °C as indicators of colitis severity [25].

### 4.5. Measurement of Colon Length and Spleen Weight

At the end of the experiment, mice were sacrificed, and the colon and spleen were carefully excised. Colon length and spleen weight were measured to assess macroscopic inflammation and splenic enlargement, respectively.

### 4.6. Measurement of Colon Inflammatory Biomarkers

Excised colon tissues were homogenized using a Biomasher Standard (TaKaRa Bio Inc., Shiga, Japan), and supernatants were collected for analysis. IL-1β, IL-6, and TNF-α concentrations were measured using ELISA kits (R&D Systems, Minneapolis, MN, USA) according to the manufacturer’s instructions [30]. Myeloperoxidase (MPO) activity was determined using a commercial assay kit (Abcam, Cambridge, UK; Catalog No. ab105136), and the results were expressed as units of MPO activity per milligram of tissue protein [31].

### 4.7. Western Blot Analysis

Colon tissues from two mice were pooled to obtain sufficient protein for Western blot analysis. Three independent pooled samples per group were prepared and analyzed (*n* = 3). The samples were homogenized in radioimmunoprecipitation (RIPA) buffer, and proteins were mixed with SDS sample buffer and denatured at 95 °C for 5 min. Samples were separated on 10% SDS-PAGE gels and transferred to polyvinylidene difluoride (PVDF) membranes. After blocking with 5% skim milk in tris-buffered saline with tween-20 (TBST), membranes were incubated overnight at 4 °C with primary antibodies against β-actin, COX-2, NF-κB p65, and phospho-NF-κB p65 (Cell Signaling Technology, Danvers, MA, USA; catalog numbers 4970, 12282, 4764 and 3033, respectively; 1:1000) [32]. After TBST washing, membranes were incubated with horseradish peroxidase (HRP)-conjugated secondary antibodies (1:5000). Signals were detected using an ECL substrate, and band intensities were quantified with ImageJ software (version 1.53; NIH, Bethesda, MD, USA) [26]. Cox-2 signals were normalized to the corresponding β-actin signal, whereas phosphorylated NF-κB p65 signals were normalized to the corresponding total NF-κB p65 signals from matched experimental replicates.

### 4.8. Statistical Analysis

All statistical analyses were performed using GraphPad Prism software (version 5.0; GraphPad Software Inc., La Jolla, CA, USA). Data are presented as the mean ± standard deviation (SD). Normality was assessed using the D’Agostino-Pearson omnibus test prior to statistical analysis. Body weight, water intake, and DAI scores were analyzed using two-way repeated measures ANOVA followed by Tukey’s multiple comparisons test. Other data were analyzed using one-way ANOVA followed by Tukey’s multiple comparisons test. Exact *p*-values are reported where applicable, and *p* < 0.05 was considered statistically significant.

## 5. Conclusions

The anti-inflammatory effects of J2H-1802 in DSS-induced colitis are associated with suppression of inflammatory cytokine production and inhibition of the NF-κB/COX-2 signaling pathway. These findings suggest that J2H-1802 modulates inflammatory responses at the molecular level and may support further investigation of this hybrid compound in IBD.

## Figures and Tables

**Table 1:** Disease Activity Index (DAI).

Score	Stool Consistency	Rectal Bleeding
0	Normal	Normal
1	Loose stool	Blood traces in stool
2	Mild diarrhea	Mild bleeding
3	Severe diarrhea	Severe bleeding

## Data Availability

The original contributions presented in this study are included in the article/Supplementary Material. Further inquiries can be directed to the corresponding authors.

## Supplementary Materials

The following supporting information can be downloaded at: https://www.mdpi.com/article/10.3390/ph19060847/s1, Figure S1: ^1^H NMR spectrum of J2H-1802; Figure S2: Mass spectrum of J2H-1802; Figure S3: HPLC chart of J2H-1802.

## Abbreviations

The following abbreviations are used in this manuscript:

UC	Ulcerative Colitis
DSS	Dextran Sulfate Sodium
NF-κB	Nuclear Factor kappa-light-chain-enhancer of activated B cells
COX-2	Cyclooxygenase-2
DAI	Disease Activity Index
TNF-α	Tumor Necrosis Factor-α
IL-1β	Interleukin-1beta
IL-6	Interleukin-6
MPO	Myeloperoxidase
p-NF-κB	Phosphorylated NF-κB
IBD	Inflammatory Bowel Disease
