# Supplementary material for: Design and Preliminary In Vivo Evaluation of J2H-1802, a Hybrid Compound Derived from 5-ASA and MMF, in a DSS-Induced Colitis Mouse Model
Source: Pharmaceuticals (Basel). 2026 May 29;19(6):847. doi: 10.3390/ph19060847 (PMC13305943; doi:10.3390/ph19060847)
Supplement: Supplementary file 1 [file pharmaceuticals-19-00847-s001.zip › pharmaceuticals-4308771-supplementary.pdf]

Supplementary material

Design and preliminary in vivo evaluation of J2H-1802, a hybrid compound derived from 5-ASA and MMF, in a DSS-induced colitis mouse model

Myong Jin Lee[1,†], Sung-Hoon Park[2,†], Gabsik Yang[1], Jason Kim[3], Ju Young Lee[3], Kwanghyun Choi[3], Kiwon Jung[3,4], Ji Hwan Lee[1], Sumi Lee[5], Woo-Chan Son[6,*], and Ki Sung Kang[1,*]

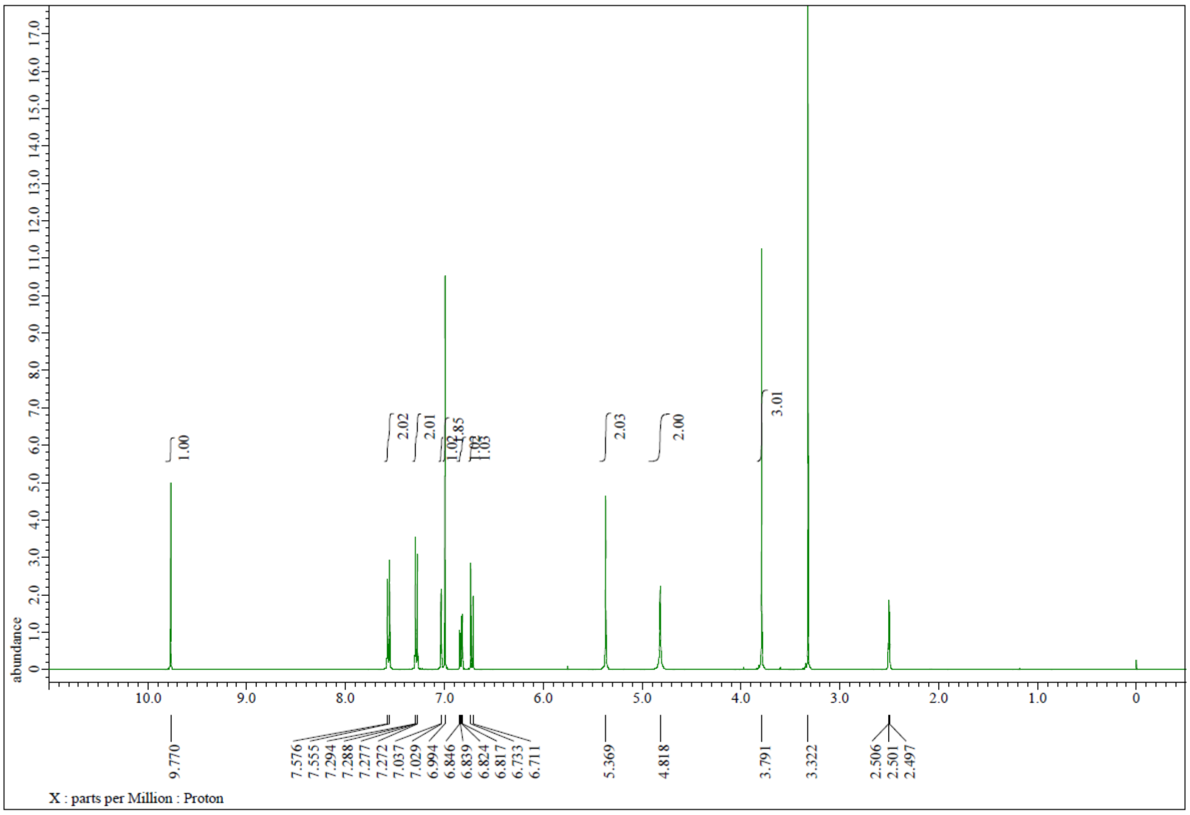

Figure S1. ^{1}H NMR spectrum of J2H-1802 (400 MHz, solvent: DMSO-*d6*)

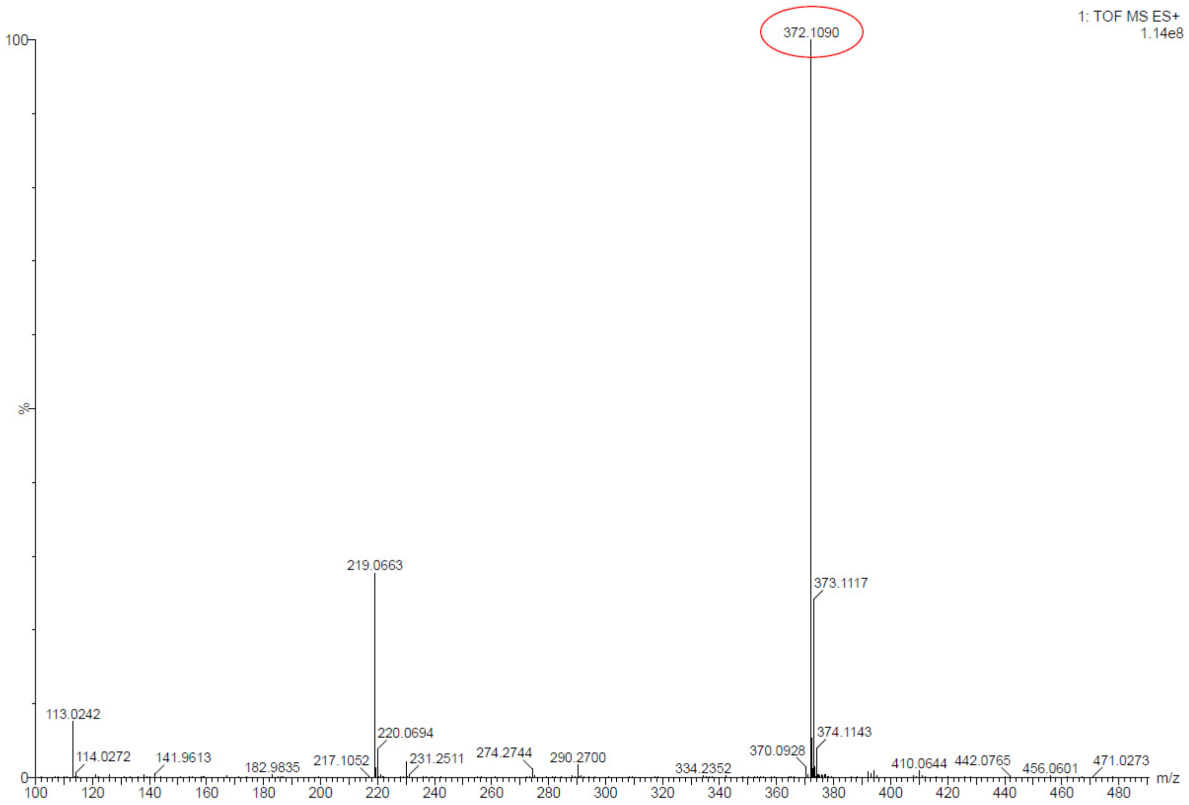

Figure S2. Mass spectrum of J2H-1802 (ES+)

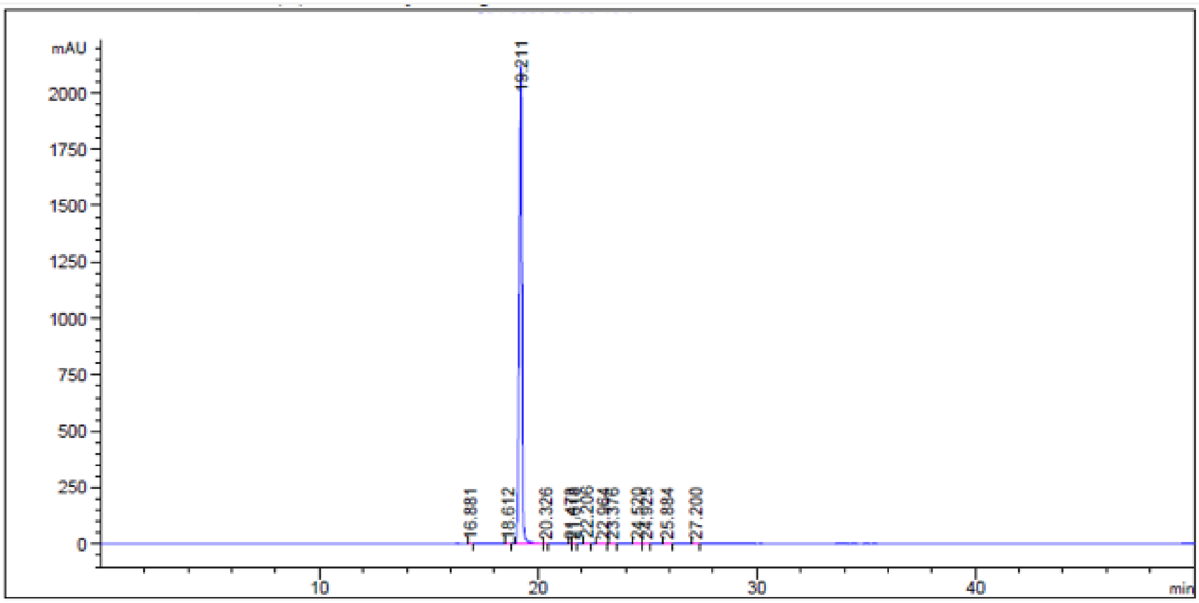

Area Percent Report

Sorted By : Signal
Multiplier : 1.0000
Dilution : 1.0000
Do not use Multiplier & Dilution Factor with ISTDs

Signal 1: VWD1 A, Wavelength=235 nm

Peak #	RetTime [min]	Type	Width [min]	Area [mAU*s]	Height [mAU]	Area %
1	16.881	BB	0.0698	10.12508	2.19697	0.0572
2	18.612	BBA	0.1027	1.84274	2.73135e-1	0.0104
3	19.211	BBA	0.1293	1.76128e4	2112.46924	99.5356
4	20.326	BB	0.1201	2.14385	2.83842e-1	0.0121
5	21.473	BBA	0.0882	2.20046	3.99993e-1	0.0124
6	21.618	BBA	0.0836	2.26204	4.43121e-1	0.0128
7	22.206	BBA	0.1214	19.22101	2.40031	0.1086
8	22.964	BBA	0.1119	14.64117	2.03505	0.0827
9	23.376	BB	0.1089	2.27503	3.27969e-1	0.0129
10	24.520	BB	0.1165	10.62424	1.46627	0.0600
11	24.925	BB	0.1171	2.78022	3.64086e-1	0.0157
12	25.884	BB	0.1206	2.72773	3.43750e-1	0.0154
13	27.200	BBA	0.1117	11.33354	1.65942	0.0640

Figure S3. HPLC chart of J2H-1802
